# Neochord DS1000 system versus conventional mitral valve repair for correction of mitral regurgitation due to prolapse of the posterior leaflet

**DOI:** 10.1093/icvts/ivac139

**Published:** 2022-05-27

**Authors:** Tirone E David

**Affiliations:** Division of Cardiovascular Surgery, Peter Munk Cardiac Centre, Toronto General Hospital, University of Toronto, Toronto, ON, Canada

**Keywords:** Gore-Tex chord, Mitral valve repair, Mitral regurgitation

In this issue of the *Interactive CardioVascular and Thoracic Surgery*, D’Onofrio and colleagues [1] from the University of Padua compared the perioperative and 5-year outcomes of correction of mitral regurgitation (MR) due to posterior leaflet prolapse with Neochord DS1100 (NeoChord, Inc. St. Louis Park, MN, USA) versus conventional mitral valve repair. Patients were enrolled from 2010 to 2018 and after exclusions, the authors had 281 patients available for the study: 169 had Neochord and 112 had surgical repair, suggesting the authors’ preference for Neochord to treat posterior leaflet prolapse. To compensate for discrepancies procedure selection, the authors used a propensity score analysis using preoperative clinical variables believed to affect outcome. They identified 88 pairs of patients with similar clinical profiles except for functional class which was worse in the Neochord group. The patients in this study were younger than in most reports on longitudinal outcomes of mitral valve repair [2, 3]. The chose patients’ survival as the primary endpoint of the study but since a propensity score analysis was used to compensate for differences in age and comorbidities, this does not seem rational. The secondary endpoints of reoperation on the mitral valve and freedom from moderate or severe MR are far more pertinent in this type of comparing procedures to treat MR. Before discharge from hospital, 8 patients in the Neochord and 1 patient in the surgical repair group had moderate or severe MR. The 5-year survival was similar, as one would expect, but the freedom from recurrent MR was only 57.6% in the Neochord group and 84.6% in the surgical repair group, and the freedom from reoperation was 78.9% in the first group and 92% in the second.

Neochord DS1100 gained European market approval in 2016 and FDA approval for a randomized clinical trial in the USA in the same year [4]. I have not been able to find any publication on that randomized trial, likely because it is still enrolling patients in spite of the fact that 6 years have passed since its FDA approval [4]. Hopefully, this randomized clinical trial will help us to determine the usefulness of this device. Based on D'Onofrio and colleagues [1] experience and on that of others [3], I am not sure this device should be used in any patient who can have conventional mitral valve repair. There are, however, patients with posterior leaflet prolapse with intractable heart failure that would not tolerate a median sternotomy or even a right mini-thoracotomy with cardiopulmonary bypass and, perhaps, they should be considered for this device but only after excluding the feasibility of a transcatheter Mitraclip™ (Abbott Laboratories, Chicago, IL, USA), which is likely to provide better and safer outcomes [5].

I have been practicing cardiac surgery for over 4 decades and prospectively followed certain groups of my patients. Mitral valve surgery is one of them, and I have over 3000 mitral valve repairs in my database of prospectively followed patients. There are few cardiac procedures that restore lifespan and quality of life as well as mitral valve repair for MR due to leaflet prolapse. I have not lost a single patient during the past 2000 repairs, and in a recent report from out unit [2] the cumulative incidence of reoperation was 4.6% and the cumulative incidence of recurrent moderate or severe MR was 12.5% at 20 years. It would be very difficult for existing available technologies such as NeoChord or Mitraclip to provide similar outcomes.

## References

[1] D’Onofrio A , Mastro F, M. Nadali M, Fiocco A, Pittarello D, Aruta P et al Transapical beating heart mitral valve repair vs. conventional surgery: a propensity matched study. Interact CardioVasc Thorac Surg 2022. 10.1093/icvts/ivac053. PMC9252130 35234902

[2] David TE , David CM, Tsang W, Lafreniere-Roula M, Manlhiot C. Long-term results of mitral valve repair for regurgitation due to leaflet prolapse. J Am Coll Cardiol 2019;74:1044–53. 31439213 10.1016/j.jacc.2019.06.052

[3] Budra M , Janušauskas V, Drąsutienė A, Zorinas A, Zakarkaitė D, Lipnevičius A et al; NeoChord Research Group. Midterm results of transventricular mitral valve repair: single-center experience. J Thorac Cardiovasc Surg 2021. 10.1016/j.jtcvs.2020.12.142. 33612306

[4] Randomized Trial of the Neochord DS1000 System Versus Open Surgical Repair (ReChord). ClinicalTrial.Gov NCT02803957.

[5] Feldman T , Kar S, Elmariah S, Smart SC, Trento A, Siegel RJ et al; EVEREST II Investigators. Randomized comparison of percutaneous repair and surgery for mitral regurgitation: 5-year results of EVEREST II. J Am Coll Cardiol 2015;66:2844–54. 26718672 10.1016/j.jacc.2015.10.018

